# Health Care Utilization and Anti-Cancer Drug Expenditure for Six Solid Cancers in Korea From 2007 to 2019

**DOI:** 10.3389/fonc.2022.862173

**Published:** 2022-06-27

**Authors:** Juhee Park, Kyeongjun Moon, Dong-Sook Kim

**Affiliations:** Health Insurance Review and Assessment Service, Wonju, South Korea

**Keywords:** cancer, cancer therapeutics, utilization, expenditure, six solid cancer

## Abstract

**Background:**

The burden of care continues to rise considerably worldwide and the challenge of diversity in cancer research has become important. We aimed to examine trends of cancer care utilization and anti-cancer medication among patients with six solid cancers (gastric, colorectal, liver, lung, breast, and prostate cancer) in South Korea.

**Methods:**

This study analyzed patients diagnosed with six types of solid cancer from 2007 to 2019 using data from the National Health Insurance claims database. We analyzed the total number of cancer cases, each patient’s length of stay (LOS) in a hospital, the number of outpatient physician visits, total medical care costs, total out-of-pocket (OOP) costs, and expenditures on anti-cancer drugs.

**Results:**

Utilization of healthcare services and spending on cancer care including anti-cancer drugs both increased in the 13-year study period. The average LOS was the highest for colorectal cancer patients at 43.5 days, and breast cancer patients had the highest average number of physician visits at 11.8. Breast cancer patients had the highest total medical costs (USD 923 million), anti-cancer drug spending (USD 156 million), and the largest increase (5 times) over the 13-year period. The anti-cancer drugs with the largest market shares were ramucirumab for gastric cancer; oxaliplatin for colorectal cancer; sorafenib for liver cancer; pembrolizumab, nivolumab, for lung cancer; trastuzumab for breast cancer; and bicalutamide for prostate cancer.

**Conclusion:**

This study was a large-scale analysis from a nationally representative database of the total population. The study also shows the pattern of cancer care in an Asian country and can provide implications for future cancer research.

## Introduction

Cancer incidence and mortality are rapidly growing worldwide, and the global cancer burden as of 2020 has risen to 19.3 million cases with 9.9 million recorded cancer deaths (1). Despite advances in cancer care, cancer was still the second leading cause of death worldwide in 2018 (2). The highest number of new cancer cases in 2020 were breast cancer, followed by lung, colon and rectum, prostate, skin, and gastric cancer (2.26, 2.21, 1.93, 1.41, 1.20, and 1.09 million cases, respectively). The most common cause of cancer death in 2020 was lung cancer, followed by colon and rectum, liver, stomach, and breast cancer (1.80, 0.93, 0.83, 0.77, and 0.69 million deaths, respectively). To reduce cancer mortality, the World Health Assembly passed a resolution on cancer prevention and control in the context of an integrated approach (WHA70.12) in 2017 (3). In response to this action, the World Health Organization (WHO) has begun to monitor the cancer burden. However, access to cancer clinical trials of non-white racial groups remains one of the challenges of diversity to address in cancer care and biomedical research (4).

The incidence and mortality rates of cancer have increased over time in South Korea (hereafter, Korea), and cancer is regarded as one of the nation’s most significant health problems (5). Cancer is the leading cause of death in Korea, with 248,837 newly diagnosed cancer cases and 79,153 cancer deaths in 2018 (6). Also, Korea spent $2.9 billion (4.9% of healthcare expenditures) on cancer patients, who accounted for 0.6% of the total Korean population in 2018 (7).

Global spending for all medicines used to treat cancer patients increased from USD 91 billion in 2012 to USD 150 billion in 2018, driven by therapeutic drugs (8). The availability of anti-cancer medication has increased greatly, and the average annual cost of new oncology medicines continues to show an upward trend. Between 2014 and 2018, 57 new oncology therapeutics cancer drugs for 89 indications launched received approval. Novel cancer medicines (particularly targeted therapies and immunotherapies) have recently revolutionized treatment for several cancers, including immuno-oncology agents, which upended the existing therapeutic paradigm (9). In particular, programmed cell death protein 1 (PD-1) and programmed cell death-ligand 1 (PD-L1) inhibitors were quickly adopted after showing remarkable success at targeting multiple cancers.

Previous studies have estimated trends associated with expenditures related to cancer care and cancer drugs (10–12); however, there have been few studies examining the magnitude of cancer care utilization and spending related to anti-cancer therapy. In Korea, several studies have analyzed cancer incidence, survival, prevalence, and mortality using the National Cancer Incidence Database (6, 13–23), but few of these studies examined trends related to anti-cancer therapy.

The purpose of this study, therefore, was to examine the utilization of cancer care [measured using a patient’s length of stay (LOS) at hospitals and number of visits to an outpatient physician] and expenditures related to cancer care [total medical care costs and out-of-pocket (OOP) expenses] for six different types of cancer using the National Health Insurance (NHI) database.

## Methods

### Data Source

This was a retrospective cohort study that examined data from patients with six different types of solid cancer. We used NHI claims data that covered the entire population of Korea (about 51.8 million people). The NHI claims database includes data related to all ambulatory care, inpatient services, procedures, and prescriptions administered at all medical institutions and pharmacies in Korea. It includes information on the demographic characteristics of patients, patients’ disease codes, the characteristics of medical institutions, healthcare service utilization, medicine use, and medical expenditures.

### Study Population

The study population consisted of cancer patients from 2007 to 2019 whose primary or secondary disease codes corresponded to one of the six types of cancer selected for this study. The diagnostic terms used in this study were from the 10th revision of the International Statistical Classification of Diseases and Related Health Problems (ICD-10). The disease codes were as follows: gastric cancer, C16; colorectal cancer, C18-C20; liver cancer, C22; lung cancer, C34; breast cancer, C50; and prostate cancer, C61.

We used a list of medicines that were eligible for reimbursement by the Health Insurance Review and Assessment Service. Therapeutic subgroups were classified according to the Anatomical Therapeutic Chemical (ATC) classification system of the WHO Collaborating Center (24). Based on the WHO ATC classification, drugs were selected if their ATC-2 classification indicated that they were antineoplastic agents (L01) or endocrine therapy (L02). We classified drugs based on the 5-level ATC system (therapeutic class).

### Outcome Meausres and Variables

The outcome measures were the trends related to disease prevalence, the characteristics of medical institutions, the LOS of patients, the number of outpatient physician visits made by patients, medical expenses, OOP expenses, the main active ingredients in anti-cancer medications, and the cost of medications. The total medical cost was considered using the yearly increase of the consumer price index as of 2019.

The analytical dimensions were age, sex, and drug classification based on the ATC system. Prevalence was the number of patients per 1,000 people and was calculated as the number of cancer patients divided by the number of people in the total population of the same age and year taken from Statistics Korea. The ages of patients were divided into the following age groups: <65, ≥65 years old. Healthcare utilization was classified as inpatients and outpatients. The types of medical facilities were tertiary hospitals, secondary hospitals, primary hospitals, and clinics.

In this study, patients were the units of analysis. Portions of patients’ personal identification numbers were codified and blocked out to protect their privacy, and the authors were blinded to each patient’s full personal identification number. In accordance with the Declaration of Helsinki, institutional review board approval procedures were followed internally.

### Statistical Analysis

We calculated the outcome measures for each person according to the year and type of cancer. SAS Enterprise version 7.1 (SAS Institute, Cary, NC, USA) was used for all analyses.

## Results

### The Prevalence of Six Types of Cancer Among Patients

The total number of patients with one of the six different types of cancer (stomach, colorectal, lung, liver, breast, or prostate cancer) increased from 472,457 in 2007 to 880,110 in 2019. In 2019, the rate of cancer incidence per 1,000 people was the highest for breast cancer, at 4.2 (8.3 for women), followed by stomach cancer at 3.5, colorectal cancer at 3.2, prostate cancer at 2.3 (4.6 for men), lung cancer at 2.2, and liver cancer at 1.8. Stomach cancer was the most common cancer among men.

The prevalence of breast cancer increasd by 2.6 times in the 13-year study periods. Among patients who were younger than 65 years of age, the most common cancer was breast cancer, followed by stomach cancer. Among patients who were 65 years old and above, the most common cancer was prostate cancer, followed by stomach cancer, and colorectal cancer (**Table 1**).

**Table 1:** Trends in the cancer prevalence rate by cancer site, sex, and age (no. of patients per 1,000 people).

	2007	2008	2009	2010	2011	2012	2013	2014	2015	2016	2017	2018	2019
Stomach cancer													
Total	2.4	2.6	2.8	3.0	3.0	3.1	3.2	3.2	3.3	3.4	3.4	3.5	3.5
Men	3.2	3.4	3.7	4.0	4.0	4.1	4.2	4.3	4.4	4.5	4.6	4.7	4.7
Women	1.7	1.8	1.9	2.0	2.0	2.1	2.1	2.1	2.2	2.2	2.2	2.3	2.3
<65 years	0.7	0.7	0.8	0.8	0.8	0.8	0.8	0.8	0.8	0.9	0.9	0.9	0.8
≥65 years	9.6	9.9	10.4	10.9	10.6	10.6	10.6	10.5	10.3	10.3	10.1	9.9	9.7
Colorectal cancer													
Total	2.0	2.0	2.2	2.4	2.5	2.7	2.8	2.9	3.0	3.1	3.1	3.2	3.2
Men	2.3	2.3	2.6	2.8	3.0	3.2	3.4	3.5	3.5	3.7	3.7	3.8	3.8
Women	1.8	1.7	1.8	2.0	2.1	2.2	2.3	2.3	2.4	2.5	2.5	2.5	2.6
<65 years	0.6	0.5	0.6	0.6	0.6	0.7	0.7	0.7	0.7	0.7	0.7	0.7	0.7
≥65 years	8.3	8.1	8.8	9.4	9.6	10.0	10.1	10.1	10.1	10.0	9.9	9.6	9.4
Liver cancer													
Total	1.5	1.3	1.3	1.4	1.4	1.5	1.5	1.6	1.6	1.7	1.7	1.8	1.8
Men	2.1	1.9	1.9	2.0	2.1	2.1	2.2	2.3	2.4	2.5	2.5	2.6	2.6
Women	0.9	0.8	0.8	0.8	0.8	0.8	0.8	0.9	0.9	0.9	0.9	1.0	1.0
<65 years	0.5	0.4	0.4	0.5	0.5	0.5	0.5	0.5	0.5	0.5	0.5	0.5	0.5
≥65 years	4.2	3.9	3.9	4.1	4.2	4.3	4.4	4.4	4.5	4.6	4.6	4.6	4.6
Lung cancer													
Total	1.2	1.1	1.2	1.3	1.3	1.4	1.5	1.6	1.7	1.8	1.9	2.0	2.2
Men	1.6	1.6	1.6	1.7	1.8	1.9	2.0	2.1	2.2	2.3	2.4	2.6	2.7
Women	0.8	0.7	0.8	0.8	0.9	1.0	1.0	1.1	1.2	1.3	1.4	1.5	1.6
<65 years	0.3	0.2	0.2	0.3	0.3	0.3	0.3	0.3	0.3	0.3	0.3	0.4	0.4
≥65 years	6.0	5.7	5.7	5.9	6.0	6.1	6.3	6.4	6.5	6.7	6.9	7.2	7.4
Breast cancer													
Total (based on total population)	1.6	1.7	1.9	2.1	2.2	2.4	2.6	2.8	3.0	3.3	3.6	3.9	4.2
Women	3.2	3.5	3.9	4.2	4.5	4.8	5.2	5.6	6.0	6.6	7.1	7.7	8.3
<65 years	3.1	3.4	3.7	4.1	4.4	4.7	5.0	5.4	5.8	6.3	6.8	7.4	7.9
≥65 years	3.9	4.3	4.8	5.2	5.5	6.0	6.5	7.0	7.5	8.1	8.9	9.6	10.5
Prostate cancer													
Total based on total population	0.9	0.7	0.8	0.9	1.0	1.2	1.3	1.4	1.6	1.7	1.9	2.1	2.3
Men	1.8	1.4	1.7	1.8	2.0	2.3	2.6	2.8	3.1	3.5	3.8	4.2	4.6
<65 years	0.7	0.4	0.5	0.5	0.5	0.6	0.6	0.7	0.7	0.8	0.8	0.9	1.0
≥65 years	15.0	12.9	14.9	15.9	17.6	19.3	20.8	21.6	23.2	24.9	26.2	27.7	28.9

### Trends Related to Cancer Care Utilization

**Table 2** shows the utilization of healthcare services and medical spending of patients. Breast cancer was the most prevalent cancer type, with 215,393 cases in 2019, and showed the largest average increase across the 13-year study period at 14.5%, followed by stomach cancer, colorectal cancer, prostate cancer, lung cancer, and liver cancer.

**Table 2:** Trends in healthcare utilization and spending by cancer type and year.

	2007	2008	2009	2010	2011	2012	2013	2014	2015	2016	2017	2018	2019	Average growth rate (%)
Stomach cancer														
No. of patients	119,058	127,934	138,792	149,607	151,185	155,505	160,345	163,954	167,013	172,674	175,013	177,409	179,511	(4.2)
Length of admission per patient	28.7	29.5	29.1	29.8	29.9	30.0	30.4	30.8	32.0	31.9	33.0	33.5	33.8	
Outpatient visit days per patient	7.1	7.1	7.2	7.1	6.9	6.9	6.7	6.5	6.3	6.4	6.4	6.4	6.4	
Total medical cost (million $)	291	322	348	372	384	385	385	396	406	448	485	522	561	(7.7)
Medical cost considering inflation	372	392	413	429	425	417	412	419	426	465	494	524	561	(4.3)
Anti-cancer drug cost (million $)	35	32	32	31	32	30	31	31	30	29	27	36	44	(2.2)
Surgery	5	13	16	18	19	18	18	21	24	26	25	31	37	(59.3)
Radiation	1	2	2	2	3	3	3	3	3	4	4	4	4	(16.7)
Colorectal cancer														
No. of patients	100,383	98,787	110,082	119,745	127,549	135,828	142,231	146,438	150,917	156,336	159,110	161,881	164,683	(5.3)
Length of admission per patient	25.2	30.7	38.7	39.7	33.8	35.6	36.1	39.9	39.8	40.5	42.2	43.4	43.5	
Outpatient visit days per patient	7.0	8.1	10.0	11.0	7.9	8.0	8.5	9.6	9.0	8.2	8.3	8.4	8.5	
Total medical cost (million $)	309	349	402	443	480	485	473	530	593	690	763	818	882	(15.4)
Medical cost considering inflation	394	425	477	510	532	526	506	560	622	717	778	821	882	(10.3)
Anti-cancer drug cost (million $)	56	59	63	65	68	60	49	73	102	127	137	138	142	(12.7)
Surgery	12	13	18	21	23	25	24	28	34	36	37	43	48	(24.7)
Radiation	9	9	11	13	15	16	15	17	20	26	27	28	31	(20.4)
Liver cancer														
No. of patients	73,537	66,022	66,887	69,536	71,140	74,104	77,683	79,854	83,464	86,571	87,185	90,319	91,222	(2.0)
Length of admission per patient	28.1	30.0	29.1	29.3	30.0	29.9	30.2	30.2	29.4	30.0	30.2	30.2	30.0	
Outpatient visit days per patient	7.2	8.3	8.8	9.0	9.2	9.3	9.1	9.1	9.0	9.1	9.2	9.2	9.4	
Total medical cost (million $)	250	285	310	343	372	396	405	422	445	490	522	559	601	(11.7)
Medical cost considering inflation	318	347	367	395	412	429	433	446	467	509	532	561	601	(7.4)
Anti-cancer drug cost (million $)	3	3	3	3	4	5	9	8	8	7	7	10	10	(24.8)
Surgery	2	3	3	4	4	5	5	6	8	8	8	11	12	(33.0)
Radiation	4	6	7	7	8	9	9	10	14	17	19	21	23	(35.3)
Lung cancer														
No. of patients	58,078	56,636	58,703	63,048	66,496	71,090	75,247	79,916	84,372	90,942	97,014	104,668	112,093	(7.8)
Length of admission per patient	34.2	37.2	36.3	37.1	38.0	37.9	38.2	38.4	37.4	37.7	38.6	38.2	37.5	
Outpatient visit days per patient	10.4	11.3	11.7	11.7	11.8	11.7	11.4	11.4	11.2	11.2	11.3	11.6	11.7	
Total medical cost (million $)	276	319	342	383	408	414	428	466	493	569	656	801	894	(18.7)
Medical cost considering inflation	352	389	405	442	452	449	458	492	517	590	668	804	894	(12.8)
Anti-cancer drug cost (million $)	60	70	71	76	73	70	69	68	66	57	66	126	140	(11.2)
Surgery	4	4	7	10	11	12	13	16	20	23	26	34	38	(80.8)
Radiation	13	15	18	21	24	26	28	33	40	50	58	66	74	(38.5)
Breast cancer														
No. of patients	78,707	86,833	96,021	105,560	113,236	122,225	132,070	141,954	153,196	168,498	183,818	199,652	215,393	(14.5)
Length of admission per patient	27.8	29.6	29.6	30.6	32.7	35.1	36.7	37.6	37.5	37.6	38.3	37.8	38.5	
Outpatient visit days per patient	11.9	12.3	12.5	12.7	12.7	12.7	12.4	12.3	12.1	12.3	12.0	11.9	11.8	
Total medical cost (million $)	183	216	259	313	370	399	419	469	510	598	684	804	923	(33.7)
Medical cost considering inflation	233	263	307	361	409	432	448	495	535	621	696	807	923	(24.7)
Anti-cancer drug cost (million $)	42	47	56	81	105	113	109	118	125	88	82	119	156	(23.0)
Surgery	1	1	1	1	1	1	1	1	2	2	13	34	37	(475.4)
Radiation	19	22	27	31	37	43	48	55	69	88	99	115	127	(48.5)
Prostate cancer														
No. of patients	43,187	35,475	41,888	45,143	51,105	58,327	66,550	71,754	79,052	88,535	97,161	108,081	118,124	(14.5)
Length of admission per patient	19.1	22.4	22.0	23.2	23.8	25.6	26.9	27.0	28.2	26.1	26.3	25.9	26.3	
Outpatient visit days per patient	6.0	7.9	7.9	8.3	8.4	8.2	7.7	7.5	7.1	7.3	7.4	7.5	7.7	
Total medical cost (million $)	50	60	70	80	97	108	116	125	137	163	194	232	274	(36.9)
Medical cost considering inflation	64	73	83	92	107	117	124	132	144	169	198	233	274	(27.2)
Anti-cancer drug cost (million $)	14	16	18	20	21	21	22	22	23	25	26	29	32	(10.4)
Surgery	0	0	0	0	0	0	0	0	0	0	0	0	1	(34.4)
Radiation	4	4	4	5	10	17	18	20	23	30	35	43	52	(110.8)

Healthcare utilization per patient, total medical spending, and anti-cancer drug expenditures also all increased. The average LOS per patient was the highest for colorectal cancer at 43.5 days, followed by breast cancer at 38.5 days and lung cancer at 37.8 days. The average number of physician visits was the highest for breast cancer patients at 11.8, followed by lung cancer at 11.7. Total medical costs and anti-cancer drug spending were the highest for breast cancer at USD 923 million and USD 156 million, respectively, followed by lung cancer at USD 894 million and USD 140 million. The total medical costs for prostate cancer were the lowest at USD 232 million USD, and anti-cancer drug spending was the lowest for liver cancer at USD 10 million. Total medical expenditures increased the most for prostate and breast cancer, while anti-cancer drug spending increased the most for liver and breast cancer. The cost of surgery was the highest for colorectal cancer at 48 million USD, followed by lung cancer, stomach cancer, and breast cancer at 37 million USD.

**Figure 1:**
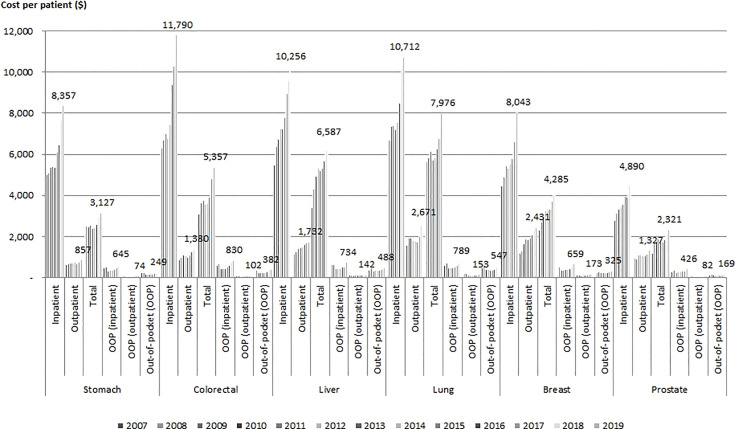
Trends in total healthcare spending by cancer type and year (USD).

**Figure 1** shows the medical costs and OOP costs per patient by type of cancer. The average inpatient cost for treatment in 2019 was the highest for colorectal cancer patients at USD 11,790 (with an average annual increase of 7.9%), followed by lung cancer at USD 10,712, and it was the lowest for prostate cancer at USD 3,933. The average total cost of outpatient physician visits in 2019 was the highest for lung cancer at USD 2,671, followed by breast cancer at USD 2,431. The annual cost of cancer care per capita ranged USD 2,321 for prostate cancer to USD 7,976 for lung cancer. The average annual increase in inpatient costs and outpatient costs were the highest for colorectal cancer at 7.9%, breast cancer at 9.0%, and stomach cancer at 48.6%, followed by liver cancer at 48.3%.

Colorectal cancer had the highest average inpatient OOP cost per patient in 2019 at USD 830, followed by lung cancer at USD 789. Breast cancer had the highest average outpatient OOP cost per patient at USD 173, also followed by lung cancer at USD 153. The increases in both the inpatient and outpatient OOP costs per patient were lower than the annual growth rate of total medical expenditures per patient. The annual growth rate of outpatient OOP costs decreased for lung cancer.

### Anti-Cancer Drugs With the Largest Market Shares

**Figure 2:**
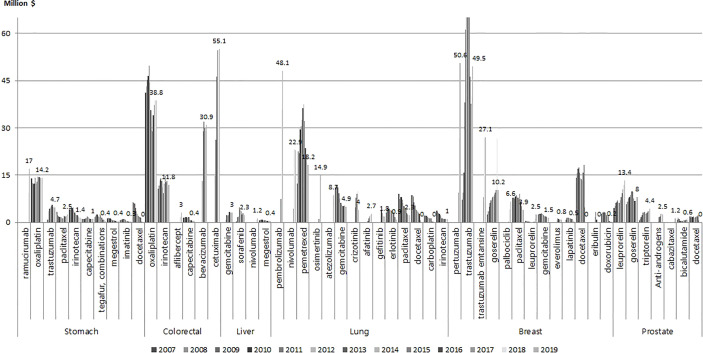
Market share of anti-cancer drugs by cancer type and year (million USD).

**Figure 2** shows the anti-cancer drugs with the largest market shares for each of the six types of cancer. For stomach cancer, the drugs with the largest market share in 2019 were ramucirumab and oxaliplatin at 17 million USD and 14.2 million USD, and the market share of ramucirumab showed a sharp increase since its introduction in 2018. For colorectal cancer, cetuximab and oxaliplatin showed the highest market share at 55.1 million USD and 38.8 million USD, respectively in 2019. For lung cancer pembrolizumab at 48.1 million USD, nivolumab at 22.9 million USD, pemetrexed, gefitinib, and erlotinib had the highest market share. For breast cancer, pertuzumab, trastuzumab, and trastuzuma emtasine had the highest market share at 50.6 million USD, 49.5 million USD, and 27.1 million USD, respectively. For prostate cancer, leuprorelin and goserelin had the highest market share.

## Discussion

The present study is the first to analyze trends in the utilization of cancer care and expenditures for six different types of solid cancers among the entire national population of Korea over a long period of time (13 years). Our study showed that total medical costs and anti-cancer drug spending related to treatment for the six selected cancer types increased by 2-3 times, from USD 1.4 billion and USD 0.2 billion, respectively, in 2007 to USD 4.2 billion and USD 0.4 billion in 2019. Although the cost per cancer patient has increased, the proportion of anti-cancer drugs relative to the overall medical costs related to cancer care decreased from 2007 to 2019. Our finding indicate that annual cost of cancer care excluding anti-cancer therpy cost per capita ranged USD 2,321 for prostate cancer to USD 7,976 for lung cancer.

The prevalence rates of the selected cancer types showed being somewhat different from the global cancer trend (25), however, these were consistent with a previous study that stomach cancer had the highest prevalence rate for both sexes in Korea (6). This pattern likely occurred due to the cancer incidence rate in Korea being somewhat different from other countries (25).

Depite limitation in direct comparison, in our study, total medical costs for the six selected cancer types increased more than doubled and this result was consistent with previous study in other countries. Several studies reported cancer-specific health expentirue in Europe. In 31 countries of Europe, the total cost of cancer care was EUR 199 billion, and expenditures almost doubled, from EUR 52 billion to EUR 103 billion, from 1995 to 2018, and the number of newly diagnosed cancer cases increased by about 50% (12). Additionally, in Europe, the proportion of cancer-specific health expenditures increased from 5.9% in 1995 to 6.2% in 2018. In 2018, the average health expenditure related to cancer care per patient was EUR 195, and the average cancer drug spending per patient was EUR 61 (12). Another study in Canada identified the growth factors related to overall cancer drug expenditures (11). In the US, cancer treatment accounts for USD 60.9 billion of direct medical costs and USD 15.5 billion of indirect morbidity costs based on data that ranged from 1998 to 2000 (10). However, a study based on literature review across 5 countries (France, Gernany, Italy, Spain, and United Kingdom) reported direct per patient expenditures was average EUR 4,966 and decreased between 2006-2015.While cancer drug spending per patient increased over the 10-year study period, and was average EUR 1,457 in 2015 (26). Even considering that the helath policy and health care environment differs from country to country, the pattern of increase in cancer care cost is common.

To the best of our knowledge, this is the first population-based study to analyze the magnitude of costs related to cancer care and expenditures related to anti-cancer drugs. To date, few large-scale analyses of the use of anti-cancer medications that cover an entire national population have been published, in either Korea or internationally. Most existing studies used the IQVIA database; thus, the estimated results used in other studies might differ from actual expenditures. This study used data from a nationally representative dataset that covered the total population of Korea, and it therefore included all recorded cancer cases in Korea.

This study examined the utilization of healthcare services by cancer patients and expenditures related to their care using an actual database that accounted for the entire national population of Korea, and the real-world findings of the present study elucidate real-life changes in cancer treatment patterns. These results could thus provide insights to aid in the development of clinical treatment guidelines in other countries. Furthermore, the LOS of patients in hospitals, number of outpatient visits, total medication expenditures (including those for outpatient visits), and drug costs by cancer type were evaluated in the present study. In addition, we analyzed patterns in anti-cancer drugs according to the market shares and we found that the use of high-cost anti-cancer drugs is rapidly increasing. These results can provide the real-world practice of anti-cancer usage, thus the need for clinical guidance to consider cost-effectiveness in order to optimize the treatment of drug therapy.

Despite increasing attention to cancer care, ethnic disparites have been coutinued to be a critical problem even in developed countires such as Europe and Untied States. Also, there is a lack of evidence in cancer care oversight among heterogenous population from Asia and Africa countires (4). This results will be important evidence of epidemiological data on cancer patients and cancer care treatement pattern.

This study also has several limitations. We included all prevalent cases in our analysis, so patients who did not utilize any healthcare services may not have been included. In addition, patients receiving treatment in the context of clinical trials were excluded. A study in the US also reported that 56% of cancer survivors were diagnosed within the past 10 years using data from the Surveillance, Epidemiology, and End Results cancer registries (27). Also, the rapid rise in total medical expenses might be caused by the increase in hospitalization costs and consultation fees, since these hospitalization fees have increased over the past decade and the coverage of health insurance has expanded. Given that cancer care is associated with high initial treatment costs, it may be more appropriate to analyze similar datasets according to the period of cancer morbidity. This study instead focused on analyzing treatment patterns on an annual basis, however, and we did not attempt to analyze the morbidity period. Further studies should therefore analyze per capita healthcare expenditures by distinguishing between initial treatment and end-of-life treatment.

In conclusion, the utilization patterns of cancer care services among cancer patients and spending related to cancer care have both increased. The market share of anti-cancer drugs for lung and breast cancer has also increased significantly due to novel drugs. Further studies are needed to provide better insight for policymakers to make efforts to improve the cost-efficiency of cancer care and the usage of new, high-cost anti-cancer drugs.

## Data Availability Statement

Data are not accessed because only those with restrictions can perform analysis at our institution. Requests to access the datasets should be directed to https://opendata.hira.or.kr/op/opc/selectOpenDataInfoView.do.

## Ethics Statement

The studies involving human participants were reviewed and approved by Institutional Review Board in Health Insurance Review and Assessment Service. Written informed consent for participation was not required for this study in accordance with the national legislation and the institutional requirements.

## Author Contributions

D-SK: Data management, analysis, and writing the draft paper. JP: Design of study, writing, and revising the manuscript. KM: Data management. All authors contributed to the article and approved the submitted version.

## Conflict of Interest

The authors declare that the research was conducted in the absence of any commercial or financial relationships that could be construed as a potential conflict of interest.

## Publisher’s Note

All claims expressed in this article are solely those of the authors and do not necessarily represent those of their affiliated organizations, or those of the publisher, the editors and the reviewers. Any product that may be evaluated in this article, or claim that may be made by its manufacturer, is not guaranteed or endorsed by the publisher.
